# Point Prevalence Survey of Antibiotic Use in Latin American Hospitals: 2022–2023

**DOI:** 10.3390/antibiotics14111078

**Published:** 2025-10-27

**Authors:** Paola Lichtenberger, Gabriel Levy-Hara, Robin Rojas-Cortés, Tatiana Orjuela, Jose Pablo Diaz-Madriz, Pilar Ramon-Pardo, Jose Luis Bustos, Anahí Dreser, Tania Herrera, Marcela Pilar Rojas-Diaz, Giovanna Huaquipaco, Didia Sagastume, Jose Luis Castro, on behalf of the Latin American PPS Group

**Affiliations:** 1 University of Miami Miller School of Medicine, Miami, FL 33136, USA; 2 Hospital de Agudos Carlos G. Durand, Buenos Aires 1405, Argentina; 3 Pan American Health Organization, Washington, DC 20037, USA; 4 Independent Consultant, Bogotá 20037, Colombia; 5 Pharmacy Department, Clínica Bíblica, San José 1307-1000, Costa Rica; 6 Independent Consultant, Guatemala City 01009, Guatemala; 7 National Institute of Public Health, Cuernavaca 62100, Mexico; 8 Ministry of Health, Santiago de Chile 2025, Chile; 9 Ministry of Health and Social Protection, Bogotá 111711, Colombia; 10 General Directorate of Medicines, Supplies and Drugs, Lima 15088, Peru; 11 Independent Consultant, Buenos Aires 1058, Argentina

**Keywords:** point prevalence survey (PPS), antimicrobial stewardship, global health, antimicrobial consumption, low- and middle-income countries, Latin America

## Abstract

**Background:** Antimicrobial resistance (AMR) is a public health challenge, exacerbated by the inappropriate use of antibiotics (ABs) and the lack of standardized surveillance in healthcare settings. Objective: The Latin American PPS aimed to provide a standardized methodology for monitoring antibiotic use, gather data on antibiotic prescription practices, and support initiatives for antimicrobial stewardship (AMS). **Methodology:** Using a Spanish-adapted version of the WHO PPS methodology, a point prevalence survey (PPS) was conducted between 2022 and 2023 in 67 hospitals across five Latin American countries. **Results:** A total of 11,094 patients were surveyed, of which 47.9% received at least one AB; surgical and intensive care units displayed the highest prevalence. Most prescribed AB were third-generation cephalosporins (3GC) (22.0%), carbapenems (12.1%), glycopeptides (9.2%), and penicillin combinations (8.6%). A substantial use of agents classified under the WHO’s “Watch” group was found, with notable variances across countries. A multilevel logistic regression model identified that patient age, ICU admission, recent hospitalization, the presence of a catheter, and intubation were significantly associated with higher odds of AB use. In contrast, patients admitted to obstetric or pediatric wards had lower odds of receiving antibiotics. The model revealed considerable heterogeneity between countries, even after adjusting clinical and demographic factors. **Conclusions:** This study highlights AMS opportunities through targeted interventions, such as optimizing surgical prophylaxis, reducing the use of 3GC, carbapenems, and glycopeptides, and improving adherence to CPGs. These findings provide a comprehensive framework for policymakers and healthcare facilities to develop AMS strategies tailored to the Latin American context.

## 1. Introduction

Antimicrobial resistance (AMR) has emerged as one of the leading public health threats of the 21st century. It is estimated that AMR alone was responsible for 1.27 million deaths and contributed to 4.95 million deaths globally in 2019 [1].

AMR is a problem with many interrelated causes. Studies have reported that the inappropriate use of antimicrobials is as high as 50% in healthcare facilities [2,3]. Antimicrobial use targets have been proposed as one of several measures to curb its unnecessary use [2,3,4].

Surveillance systems are essential to guide measures aimed at preventing the dissemination of AMR. Collecting hospital data and implementing informed interventions to improve AB use in hospitals has a significant potential to lower resistance [3]. Such monitoring not only supports the optimization of therapies but also plays a crucial role in enhancing equitable access to effective treatments by informing procurement requirements, ensuring availability, and guiding national policy decisions. Targeted interventions based on surveillance data can help healthcare systems allocate resources more efficiently, reducing supply shortages and ensuring that essential ABs are accessible where they are most needed.

Continuous hospital data collection on AB prescription and use is not always possible, as it requires significant resources. An alternative is to collect data during a specific period by implementing a point prevalence survey (PPS), which is already in use in hospitals around the globe [5,6,7,8,9]. The WHO has developed a protocol that meets the needs of low- and middle-income countries (LMICs) while allowing comparability with data collected in high-income countries [3]. The WHO PPS methodology is an adaptation of the one developed by the European Centre for Disease Prevention and Control (ECDC) [5], complemented by instruments from the United States’ Centers for Disease Control and Prevention (CDC) [6], the Global PPS [7], and the Medicines Utilization Research in Africa (MURIA) group [8]. 

In 2022, the Latin American PPS, a collaborative group by the Pan American Health Organization (PAHO), government institutions, and hospitals in Latin America (LATAM), published the results of a cohort of 33 hospitals in five countries using a modified version of the WHO PPS [10].

The aim of this article is to present the results of a PPS performed after the COVID-19 pandemic, in 67 hospitals from five Spanish-speaking countries (Mexico, Chile, Peru, Colombia, and Panama) between 2022 and 2023. So far, this is the largest PPS performed in LATAM.

## 2. Methodology

The Latin American PPS used a Spanish-language modified version of the WHO/PPS methodology, where only ABs were used [3,9]. The main differences included the exclusion of the McCabe score (an optional variable in the WHO protocol) and the criteria used to assess compliance with clinical practice guidelines (CPGs) [11]. The survey instrument is available in Supplementary File S1.

### 2.1. Selection of Hospitals

A sample of hospitals was chosen by the Ministries of Health (MoHs) in Peru, Colombia, Chile, and Panama, and in Mexico, they were chosen by the National Institute of Public Health and the Ministry of Health of Mexico City, in agreement with the Latin American PPS coordination team. The selected institutions were acute hospitalization centers of the second or third level of care, with access to ethics committees for protocol approval, and were interested in implementing the survey.

### 2.2. Selection of Patients

Patients hospitalized at 8:00 am on the day of the survey, regardless of whether they received ABs or not, were included. Outpatients (e.g., same-day treatment or surgery and discharge, outpatient departments, and emergency rooms or outpatient dialysis) were excluded.

### 2.3. Training and Procedures for Data Collection 

Once the MoHs had selected hospitals and a coordinator by institution, virtual meetings were held for each country, explaining the initiative. Each hospital that agreed to participate created a work team and submitted the master protocol for approval by its corresponding ethics review committee.

Hospital teams received virtual training on how to access and complete the data collection tool, developed on the Research Electronic Data Capture (REDCap) platform. Each hospital coordinator created a timeline for the different hospital wards.

The survey was conducted over a three-week period, commencing with the survey of the first ward. All beds in each ward (e.g., general surgery) were surveyed in a single day, and each ward was studied only once during the study period.

Hospital wards included medical (MED), surgical (SUR), intensive care unit (ICU), pediatrics (PED), gynecology and obstetrics (GO), high-risk (hematology, oncology, burn, transplant, and infectious diseases) (HR), and mixed (MX) wards.

### 2.4. Variables and Antibiotics Collected

The survey was divided into two sections. The first one (patient information) was completed for all patients and included the demographics, type of ward, date of admission, catheterizations, intubations, and surgeries during the current admission and previous hospitalizations during the 90 days prior to the current admission. The second part (indication and AB data) was completed only for patients receiving ABs on the survey day. Antibiotics prescribed before the day of the survey or those prescribed but not administered the day of the survey were excluded following the PPS methodology of the PAHO [3].

All systemic ABs listed in the original WHO protocol (Anatomical Therapeutic Chemical—ATC codes J01), along with oral vancomycin (A07AA09) and metronidazole (P01AB01), were available to be ticked in a dropdown list. Topical ABs and those used to treat tuberculosis were excluded. Information requested included the type of indication (treatment or prophylaxis), guidance for treatment (empiric or tailored to microbiological findings), diagnosis, microbiological results, ABs prescribed (drug, dose, interval, and route of administration), and compliance with CPGs. A prescription was considered compliant if it was in line with local, national, or international evidence-based CPGs according to the research team and validated by the PAHO coordination team based on the PAHO manual supplemental material [11]. When the assessment of compliance was not possible (e.g., the type of indication was unknown or other than prophylaxis or treatment; or the diagnosis was unknown, undefined, or other than prophylaxis or treatment), it was classified as not assessable. 

### 2.5. Data Collection, Safety, and Review 

Data were electronically collected on REDCap, whose access and maintenance services were provided by the PAHO. Data collection was conducted by trained members of the hospital teams, who completed the survey based on medical records and clinical information. Each hospital was allocated a period of 1 to 3 weeks to complete data collection across all wards. Each hospital team member had an identifiable number assigned, in case clarification was needed during the validation process. They also had access to their hospital’s de-identified data but not to information from the other hospitals. Only the Latin American PPS coordination team had access to all anonymized patient data. 

Each hospital was assigned to a Latin American PPS coordinator to support, review, and validate the data. Only the hospital team had access to the patients’ identities. The patients’ personal data were replaced with a patient number. Data were safely stored on a server hosted by the PAHO.

### 2.6. Data Analysis

Data was analyzed using R version 4.5.0. Descriptive statistics were calculated for demographic and clinical variables. To identify factors associated with antibiotic use, a multilevel logistic regression model was applied, with the presence or absence of antibiotic prescription as the dependent variable. A random intercept for country was included to account for the hierarchical structure of the data (patients nested within countries). Univariate multilevel models were first fitted for each variable. Variables with clinical relevance or statistically significant associations in the univariate analysis were included in the final multivariate model. Odds ratios (ORs), 95% confidence intervals (CIs), and *p*-values were reported for all fixed effects. The random effect for countries was interpreted as residual variation in antibiotic use between countries, after adjusting for individual-level covariates.

## 3. Results

### 3.1. Demographic and Clinical Information of Patients Enrolled

The Latin American PPS was conducted between March 2022 and November 2023 and included 67 hospitals from 5 countries: Chile (13; *n* = 3339), Colombia (8; *n* = 1087), Mexico (22; *n* = 2292), Panama (1; *n* = 298), and Peru (23; *n* = 4078). A total of 11,094 patients were surveyed (50.7% men), regardless of whether they received antibiotics. The median age of patients who were older than 2 years was 46.7 years (Standard Deviation (SD) of 25.1), while the median age for patients under 2 years was 7.3 months (SD of 6.5). Most patients had a peripheral catheter (70.9%). Approximately 9% of the patients had a history of previous hospitalizations (*n* = 1006). Table 1 describes the demographic and clinical characteristics of patients enrolled. 

### 3.2. Antibiotic Use

**Figure 1:**
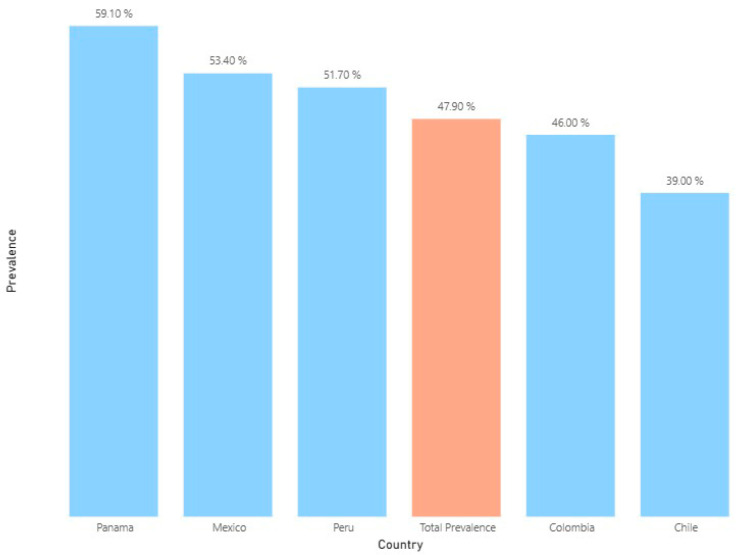
Average prevalence of antibiotic use in selected hospitals of five Latin American countries between 2022 and 2023 (n: 11,094).

A total of 5310 patients (47.9%) received at least one AB, with variations among the five countries. The lowest prevalence of ABs was found in Chilean hospitals (39.0%), and the highest prevalence was observed in the Panamanian one (59.1%) (Figure 1).

Overall, SUR, ICU, and MIX wards had the highest prevalence of AB use (56.5%, 56.0%, and 55.1%, respectively), and GO wards had the lowest prevalence (33.0%) (Table 2).

7735 AB prescriptions were identified. Third-generation cephalosporins (22.0%) were the class that was most used, followed by carbapenems (12.1%), glycopeptides (9.2%), and penicillin combinations (8.6%) (Table 3). In terms of specific ABs, ceftriaxone (17.9%), meropenem (9.3%), vancomycin (9.2%), and metronidazole (7.6%) were the most frequently prescribed. Most patients received one (29.6%, *n* = 3284) or two ABs (17.2%, *n* = 1904). Three ABs were prescribed in 1.1% (*n* = 120) cases.

### 3.3. Diagnosis Supporting the Indication for Antibiotic Prescriptions 

Antibiotic indications included 5552 diagnoses. Most ABs were used for community-acquired infections (44.6%), followed by healthcare-associated infections (31.6%), surgical prophylaxis (13.9%), and medical prophylaxis (5.7%). The most common diagnoses were pneumonia (24.0%), intra-abdominal sepsis, including hepatobiliary (14.3%), skin infections, excluding osteomyelitis (11.9%), and clinical sepsis (8.6%). Overall, the most common ABs used for these conditions were third-generation cephalosporins (24.4%), imidazole derivatives (26.3%), lacosamide’s (18.3%), and other aminoglycosides (18.6%), respectively (Table 4).

### 3.4. Compliance with Guidelines for Treatment and Prophylaxis Prescriptions

Overall, 63% of assessable prescriptions were compliant with CPGs, with higher values in Chile and Colombia (around 75%) (Table 5). Adherence to CPGs was higher in HR, ICU, and MIX wards (70–80% of prescriptions). Compliance was higher for treatment (70%) than for prophylaxis (30%). In the latter, surgical prophylaxis showed an adherence rate of 22%. 

### 3.5. AWaRe Classification of Antibiotics Prescribed

**Figure 2:**
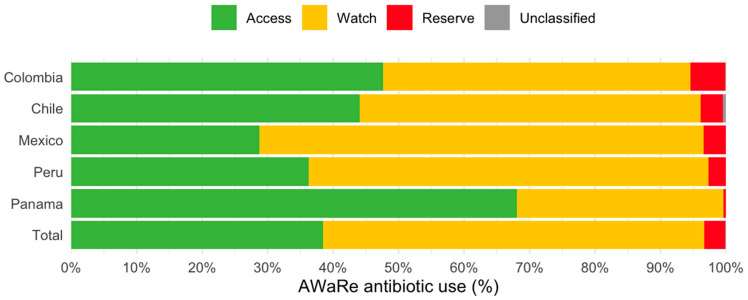
Antibiotic use according to the AWARE classification in the point prevalence survey of five Latin American countries in 2022–2023.

Figure 2 shows AB use according to WHO’s Access, Watch, and Reserve (AWaRe) classification [12]. The largest proportion corresponded to the Watch group (58.2%; *n* = 4500), followed by the Access (38.5%; *n* = 2978), Reserve (3.2%; *n* = 246), and Unclassified (0.1%; *n* = 11) groups. While Colombia, Chile, Mexico, and Peru exhibited the greatest proportion of ABs in the Watch group, the Panamanian hospital showed the largest proportions corresponding to the Access group (68.1%) and was the only one compliant with the WHO’s recommendations.

### 3.6. Factors Associated with Antibiotic Use 

A multilevel logistic regression model was used to identify factors associated with antibiotic use, accounting for the hierarchical structure of the data with a random intercept for country (Table 6). In the univariate models, patient age over one year, the male gender, previous hospitalization within 90 days, the presence of a catheter, intubation, and admission to intensive care units were associated with significantly higher odds of receiving antibiotics. Admission to adult surgical wards was also associated with higher odds, although the result narrowly missed statistical significance (*p* = 0.056). In contrast, patients admitted to pediatric wards or obstetrics and gynecology wards had significantly lower odds of antibiotic use.

In the multivariate analysis, independent predictors of higher odds of antibiotic use included patient age over one year (ORs ranging from 1.60 to 1.99 across age groups), admission to adult surgical wards (OR: 1.51; 95% CI: 1.12–2.02) or intensive care units (OR: 1.45; 95% CI: 1.05–1.99), previous hospitalization within 90 days (OR: 1.47; 95% CI: 1.27–1.69), presence of a catheter (OR: 2.71; 95% CI: 2.37–3.09), and intubation (OR: 1.53; 95% CI: 1.35–1.74). Admission to gynecology and obstetrics wards (OR: 0.65; 95% CI: 0.5–0.95) was associated with lower odds of antibiotic use. In contrast to the univariate results, no statistically significant association was found for the male gender or pediatric ward admission in the multivariate model.

The multilevel model also included a random intercept for country, which captured residual variations in antibiotic use not explained by patient-level factors. After adjusting for clinical and demographic covariates, the adjusted probability of antibiotic use varied by country. The Panamanian hospital showed the highest adjusted baseline probability (22.5%), followed by Peru (18.4%) and Mexico (16.9%). In contrast, Chile (12.5%) and Colombia (11.5%) presented lower probabilities compared to the overall average.

## 4. Discussion

Globally, nearly half of all hospital patients are prescribed antibiotics (47.70%), with prescription rates significantly higher in low- and middle-income (LMIC) economies [12,13]. A substantial proportion of these prescriptions (30–50%) are unnecessary or inappropriate, which contributes significantly to the development of antimicrobial resistance [1].

This study provides a comprehensive characterization of antibiotic prescription patterns in 67 hospitals in five countries of Latin America, including indicators reflecting the pattern and identifying its associated factors. 

This second Latin American PPS cohort revealed a regional antibiotic (AB) prescription prevalence of 47.90%, similar to that reported globally (47.70%). This figure contrasts with both the higher prevalence reported by the previous regional PPS (54.2%) and the lower rate from the Global PPS for Latin America (33.3%) [10,13,14,15,16]. The disparity is likely due to methodological differences in participant selection and sample size rather than a true directional trend [13,14,15,16], but it is still higher than the prevalence reported in HICs like Europe and North America (27–38%), suggesting significant opportunities for AB optimization in our region [5,13,14,15,16].

When comparing our findings to pre-COVID-19 data by country, we observed varying trends. While Mexico and Colombia showed a slight decrease in AB use (prevalence of 60% pre-COVID-19 to 53% in this study vs. prevalence of 52.8% pre-COVID-19 to 46%, respectively), prevalence in Peru remained stable (51% in 2019 to 48%), and Chile reported a modest increase despite maintaining the lowest prevalence in the cohort (prevalence of 35.9% in 2016 to 39% in this study) [10,13,14,15,16].

### 4.1. Antimicrobial Use Patterns

The prescription patterns observed in our study align with globally described trends, with pneumonia being the most common diagnosis (19.2%), followed by skin and soft tissue (9.8%) and intra-abdominal infections (7%) [5,13,14,15,16].

The highest AB prevalence corresponds to third-generation cephalosporins (22.0%), followed by carbapenems (12.1%), glycopeptides (9.2%), and penicillin combinations (8.6%), with ceftriaxone (17.9%) being the single product with the highest use.

Broad-spectrum cephalosporins were the preferred agent (between 22% to 27%) to treat pneumonia, intra-abdominal sepsis, and surgical prophylaxis in Chile, Mexico, Peru, and Panama. Ceftriaxone is a cornerstone of global antimicrobial therapy; however, its effectiveness is hampered by widespread misuse, leading to significant bacterial resistance [17]. Studies indicate a high prevalence of inappropriate ceftriaxone utilization, often driven by its empirical use as a first-line treatment, particularly in areas with limited access to laboratory diagnostics [18]. Factors like inadequate access to laboratory services in regions like Latin America, high bacterial resistance rates, and a lack of local antibiotic practice guidelines further exacerbate the issue [19,20]. Understanding the reasons behind the prescriber’s preference for broad-spectrum antibiotics in these regions is crucial to addressing this problem.

In our cohort, carbapenems were predominantly used for urinary tract infections (33.9%), sepsis (16.4%), and pneumonia (15%). Vancomycin was frequently administered, primarily for sepsis (18.3%) and skin and soft tissue infections (10.3%). This high utilization likely reflects the regional prevalence of ESBL-producing *E. coli* and *K. pneumoniae* (>30%), as well as the significant burden of methicillin-resistant *Staphylococcus aureus* (MRSA) in Latin America, where rates can exceed 50% in hospital-acquired bloodstream infections and community-acquired skin and soft tissue infections [19,21,22]. Even when these antimicrobials are used for an appropriate indication, their use can be further optimized. This may be achieved through evidence-based guidelines that incorporate local antimicrobial susceptibility data, advanced laboratory diagnostics, and proactive de-escalation strategies [23,24,25].

Despite similarities to Global PPS data, the AWaRe classification revealed that the use of Access antibiotics in Peru and Mexico fell significantly below the WHO’s 60% target [22]. High rates of antimicrobial resistance (AMR) could be a contributing factor, as this may limit the appropriate use of first-line, Access-group antimicrobials. A more comprehensive investigation into the interplay between antimicrobial resistance epidemiology and antimicrobial use is therefore warranted.

In Colombia and Chile, there is a relatively high use of Access-group antibiotics, while the use of Reserve-group antibiotics remains near 5% in Colombia, Mexico, and Chile [10]. These rates are among the highest in the regions studied by the Global PPS, indicating a high burden of antimicrobial resistance [10,22]. This is consistent with data from the Latin American Network for Antimicrobial Resistance Surveillance (ReLAVRA), which shows a drastic increase in carbapenem nonsusceptibility among gram-negative organisms in the region, from 0.3% in 2002 to 21% in 2016 [21].

### 4.2. Adherence to Guidelines and Influencing Factors

Our study found higher adherence to clinical practice guidelines (CPGs) for therapeutic prescriptions compared to surgical prophylaxis. This higher compliance was more pronounced in ICUs than in other wards, a finding consistent with a previous Latin American PPS [10]. Adherence to evidence-based CPGs for surgical prophylaxis was particularly low (22%), highlighting a significant need for intervention. This was notably poor in Mexico, where ceftriaxone is widely used as a prophylactic agent against current recommendations, largely due to the unavailability of cefazolin [17]. Adhering to international CPGs, which recommend using second-generation cephalosporins for less than 24 h, can optimize antimicrobial use and significantly reduce the consumption of antimicrobials in the “Watch” category [23].

The multilevel logistic regression analysis found that patients aged one year or older, patients admitted to surgical and ICU wards, those readmitted within the previous 90 days, and those with a catheter or intubation had the highest odds of receiving ABs. The model also revealed residual variability in AB use among countries, even after adjusting clinical and demographic factors, suggesting that system-level, organizational, or cultural factors may influence prescription practices. This underscores the importance of designing context-sensitive ASP interventions, aligned with national policies and hospital capacities.

### 4.3. Antimicrobial Stewardship (ASP) and National Policies

Antimicrobial stewardship programs involve a well-organized group of experts that advocate for and promote the responsible use of antimicrobials [24,25]. The PPS is a useful tool to provide ASPs with a diagnostic means to evaluate local AB prescription practices, enabling the revision of current activities and creating strategies targeting major gaps or prescription problems. It would be desirable that other countries in the region undertake these surveys to detect prevailing flaws and gaps in the use of ABs. In any case, it seems that there are common concerns that could be prioritized by health authorities in our region, including antimicrobial recommendations for pneumonia, intra-abdominal sepsis, skin infections, and surgical prophylaxis. Also, as third-generation cephalosporins, carbapenems, and vancomycin are the groups most prescribed, we recommend prioritizing their monitoring in hospitals with limited resources, where tracking or supervising all ABs is not possible, as well as encouraging the use of Access agents according to the AWaRe classification when clinically indicated and possible.

Finally, these results can support health systems in addressing access gaps, ensuring the availability of diagnostic laboratory technologies and essential ABs where they are most needed, and aligning procurement strategies with actual optimized prescription patterns. Also, it would be valuable to encourage hospitals to perform an annual PPS and repeat it periodically to have an objective measure of successful AMS implementation.

### 4.4. Study Strengths and Limitations

A key strength of this study is the large number of participating hospitals (67), surpassing previous regional efforts. This success is likely attributable to a more adaptable methodology, including an extended data collection period (three weeks), survey in Spanish and intensive training on how to use the platform. However, this study has limitations. The inability to track individual hospital and country trends across different cohorts (including the previous regional PPS) is a key constraint, as the facilities included were not identical. Despite this, this study provides a robust snapshot of AB prescription data and trends, offering valuable insights for optimization strategies across the region.

## Figures and Tables

**Table 1:** Demographic and clinical characteristics of the patients (2022–2023, n: 11,094).

	No.	%	Chile *n* (%)	Colombia *n* (%)	Mexico *n* (%)	Panama *n* (%)	Peru *n* (%)
**Number of participants**	11,094	-	3339 (30.1)	1087 (9.8)	2292 (20.7)	298 (2.7)	4078 (36.8)
**Sex**							
Male	5624	50.7	1723 (51.6)	515 (47.4)	1262 (55.1)	161 (54.0)	1963 (48.1)
Female	5453	49.2	1608 (48.2)	572 (52.6)	1028 (44.9)	137 (46.0)	2108 (51.7)
Transgender	11	0.1	6 (0.2)	0 (0.0)	2 (0.1)	0 (0.0)	3 (0.1)
Unknown	6	0.1	2 (0.1)	0 (0.0)	0 (0.0)	0 (0.0)	4 (0.1)
**Age categories**							
Less than 1 year	1487	13.4	367 (11.0)	125 (11.5)	274 (12.0)	195 (65.4)	526 (12.9)
1–4 years	679	6.1	168 (5.0)	17 (1.6)	184 (8.0)	57 (19.1)	253 (6.2)
5–17 years	1099	9.9	205 (6.1)	25 (2.3)	384 (16.8)	46 (15.4)	439 (10.8)
18–65 years	5149	46.4	1512 (45.3)	574 (52.8)	1078 (47.0)	0 (0.0)	1985 (48.7)
More than 65 years	2677	24.1	1085 (32.5)	346 (31.8)	372 (16.2)	0 (0.0)	874 (21.4)
Unknown	3	0.0	2 (0.1)	0 (0.0)	0 (0.0)	0 (0.0)	1 (0.0)
**Ward**							
Adult medical	3303	29.8	1170 (35.0)	231 (21.3)	688 (30.0)	0 (0.0)	1214 (29.8)
Pediatric	2360	21.3	494 (14.8)	63 (5.8)	672 (29.3)	229 (76.8)	902 (22.1)
Adult surgical	1792	16.2	553 (16.6)	63 (5.8)	590 (25.7)	0 (0.0)	586 (14.4)
Intensive care units	1493	13.5	607 (18.2)	205 (18.9)	205 (8.9)	69 (23.2)	407 (10.0)
Mixed	1006	9.1	111 (3.3)	406 (37.4)	38 (1.7)	0 (0.0)	451 (11.1)
Obstetrics and gynecology	893	8.0	318 (9.5)	88 (8.1)	94 (4.1)	0 (0.0)	393 (9.6)
Adult high-risk units	247	2.2	86 (2.6)	31 (2.9)	5 (0.2)	0 (0.0)	125 (3.1)
**Presence of a peripheral catheter**	7870	70.9	2167 (64.9)	964 (88.7)	1536 (67.0)	232 (77.9)	2971 (72.9)
**Patients intubated**	2153	19.4	626 (18.7)	250 (23.0)	464 (20.2)	156 (52.3)	657 (16.1)
**Previous history of hospitalization (within 90 days)**	1006	9.1	244 (7.3)	89 (8.2)	347 (15.1)	38 (12.8)	288 (7.1)

**Table 2:** Average prevalence of antibiotic use by type of hospital ward (2022–2023).

	Total		Chile		Colombia		Mexico		Peru		Panama	
	Admitted, *n*	AB Use, *n* (%)	Admitted, *n*	AB Use, *n* (%)	Admitted, *n*	AB Use, *n* (%)	Admitted, *n*	AB Use, *n* (%)	Admitted, *n*	AB Use, *n* (%)	Admitted, *n*	AB Use, *n* (%)
**Prevalence of antibiotics**	11,094	5310 (47.9)	3339	1302 (39.0)	1087	500 (46.0)	2292	1224 (53.4)	4078	2108 (51.7)	298	176 (59.1)
**Adult medical wards**	3303	1465 (44.4)	1170	429 (36.7)	231	98 (42.4)	688	346 (50.3)	1214	592 (48.8)	0	0 (0.0)
**Adult surgical wards**	1792	1012 (56.5)	553	291 (52.6)	63	40 (63.5)	590	362 (61.4)	586	319 (54.4)	0	0 (0.0)
**Intensive care units**	1493	836 (56.0)	607	274 (45.1)	205	93 (45.4)	205	144 (70.2)	407	267 (65.6)	69	58 (84.1)
**Pediatric wards**	2360	1029 (43.6)	494	156 (31.6)	63	15 (23.8)	672	282 (42.0)	902	458 (50.8)	229	118 (51.5)
**Obstetrics and gynecology**	893	295 (33.0)	318	56 (17.6)	88	24 (27.3)	94	69 (73.4)	393	146 (37.2)	0	0 (0.0)
**Adult high-risk units**	247	119 (48.2)	86	45 (52.3)	31	17 (54.8)	5	3 (60.0)	125	54 (43.2)	0	0 (0.0)
**Mixed wards**	1006	554 (55.1)	111	51 (45.9)	406	213 (52.5)	38	18 (47.4)	451	272 (60.3)	0	0 (0.0)

**Table 3:** Average frequencies of antibiotic groups prescribed by country (*n*: 7735 ^a^).

Antibiotic Group	Total, *n* (%)	Chile *n* (%)	Colombia *n* (%)	Mexico *n* (%)	Peru *n* (%)	Panama *n* (%)
J01DD Third-generation cephalosporins (ceftriaxone, cefotaxime, and ceftazidime)	1699 (22.0)	482 (25.0)	38 (5.9)	482 (26.9)	681 (21.9)	16 (6.2)
J01DH Carbapenems (meropenem, imipenem, and ertapenem)	933 (12.1)	139 7.2)	54 (8.4)	263 (14.7)	461 (14.8)	16 (6.2)
J01XA Glycopeptide antibacterials (vancomycin)	684 (8.8)	141 (7.3)	52 (8.1)	152 (8.5)	336 (10.8)	3 (1.2)
A07AA09 Antibiotics (vancomycin oral)	30 (0.4)	20 (1)	3 (0.5)	6 (0.3)	1 (0)	0 (0)
J01CR Combinations of penicillins, including beta-lactamase inhibitors (amoxicillin-sulbactam, piperacillin-tazobactam, ampicillin-sulbactam, and amoxicillin-clavulanic acid)	663 (8.6)	167 (8.7)	179 (27.8)	45 (2.5)	202 (6.5)	70 (26.9)
J01XD Imidazole derivatives (metronidazole)	587 (7.6)	276 (14.3)	13 (2)	141 (7.9)	154 (4.9)	3 (1.2)
J01FF Lincosamides (clindamycin)	480 (6.2)	73 (3.8)	42 (6.5)	99 (5.5)	255 (8.2)	11 (4.2)
J01GB Other aminoglycosides (amikacin and gentamicin)	453 (5.9)	79 (4.1)	30 (4.7)	96 (5.4)	189 (6.1)	59 (22.7)
J01DB First-generation cephalosporins (cephalexin, cefadroxil, cephradine, and cefazolin)	446 (5.8)	112 (5.8)	66 (10.2)	13 (0.7)	255 (8.2)	0 (0)
J01MA Fluoroquinolones (ciprofloxacin and levofloxacin)	392 (5.1)	78 (4)	10 (1.6)	148 (8.3)	153 (4.9)	3 (1.2)
J01CA Penicillins with an extended spectrum (ampicillin and amoxicillin)	291 (3.8)	79 (4.1)	24 (3.7)	48 (2.7)	94 (3)	46 (17.7)
J01EE Combinations of sulfonamides and trimethoprim, including derivatives (trimethoprim-sulfamethoxazole)	183 (2.4)	59 (3.1)	14 (2.2)	48 (2.7)	58 (1.9)	4 (1.5)
J01FA Macrolides (azithromycin and clarithromycin)	150 (1.9)	54 (2.8)	13 (2)	28 (1.6)	53 (1.7)	2 (0.8)
J01DE Fourth-generation cephalosporins (cefepime)	139 (1.8)	9 (0.5)	31 (4.8)	74 (4.1)	21 (0.7)	4 (1.5)
J01CF Beta-lactamase-resistant penicillins (oxacillin)	74 (1)	0 (0)	8 (1.2)	0 (0)	48 (1.5)	18 (6.9)
J01XB Polymyxins (colistin)	62 (0.8)	3 (0.2)	2 (0.3)	8 (0.4)	49 (1.6)	0 (0)
J01DC Second-generation cephalosporins (cefuroxime)	42 (0.5)	1 (0.1)	4 (0.6)	8 (0.4)	29 (0.9)	0 (0)
J01AA Tetracyclines (doxycycline)	24 (0.3)	5 (0.3)	4 (0.6)	2 (0.1)	13 (0.4)	0 (0)
J01XE Nitrofuran derivatives (nitrofurantoin)	10 (0.1)	6 (0.3)	0 (0)	2 (0.1)	2 (0.1)	0 (0)
Other antibiotics	393 (5.1)	144 (7.5) ^b^	58 (9)	127 (7.1) ^c^	59 (1.9)	5 (1.9)
Total	7735	1927	645	1790	3113	260

^a^: Number of antibiotics prescribed to 5310 patients who received at least one antibiotic during the PPS. Some patients received more than one agent. ^b^, ^c^: The category “Other antibiotics” (*n* = 393) includes agents not classified in the previous ATC groups due to low frequency. The most frequent agents in this category were as follows: for Mexico, linezolid (*n* = 48) and cephalothin (*n* = 47), and for Chile, cloxacillin (*n* = 54), linezolid (*n* = 37), and benzylpenicillin (*n* = 19).

**Table 4:** Distribution of antibiotic prescriptions by the most frequent diagnoses (*n*: 7735 ^a^).

Antibiotic Group	Pneumonia *n* (%)	Intra-Abdominal Sepsis *n* (%)	Cellulitis *n* (%)	Clinical Sepsis *n* (%)	Upper Urinary Tract Infections *n* (%)	Others *n* (%)
J01DD Third-generation cephalosporins (ceftriaxone, cefotaxime, and ceftazidime)	360 (24.4)	239 (22.9)	144 (17.9)	55 (8.4)	92 (28.0)	809 (23.6)
J01DH Carbapenems (meropenem, imipenem, and ertapenem)	225 (15.2)	135 (12.9)	74 (9.2)	108 (16.4)	111 (33.8)	280 (8.2)
J01CR Combinations of penicillins, including beta-lactamase inhibitors (amoxicillin-sulbactam, piperacillin-tazobactam, ampicillin-sulbactam, and amoxicillin-clavulanic acid)	213 (14.4)	129 (12.4)	75 (9.3)	46 (7)	24 (7.3)	176 (5.1)
J01XA Glycopeptide antibacterials (vancomycin parenteral)	137 (9.3)	59 (5.7)	83 (10.3)	120 (18.3)	6 (1.8)	309 (9.0)
J01XD/P01AB Imidazole derivatives/Nitroimidazole (metronidazol)	26 (1.8)	274 (26.3)	65 (8.1)	8 (1.2)	7 (2.1)	207 (6.0)
J01GB Other aminoglycosides (amikacin and gentamicin)	54 (3.7)	46 (4.4)	11 (1.4)	122 (18.6)	24 (7.3)	196 (5.7)
J01FF Lacosamides (clindamycin)	74 (5.0)	14 (1.3)	147 (18.3)	8 (1.2)	1 (0.3)	236 (6.9)
J01MA Fluoroquinolones (ciprofloxacin and levofloxacin)	73 (4.9)	63 (6)	52 (6.5)	7 (1.1)	19 (5.8)	178 (5.2)
J01CA Penicillins with an extended spectrum (ampicillin and amoxicillin)	45 (3.0)	24 (2.3)	6 (0.7)	120 (18.3)	5 (1.5)	91 (2.7)
J01FA Macrolides (azithromycin and clarithromycin)	100 (6.8)	3 (0.3)	1 (0.1)	1 (0.2)	1 (0.3)	44 (1.3)
J01DE Fourth-generation cephalosporins (cefepime)	44 (3.0)	5 (0.5)	9 (1.1)	18 (2.7)	4 (1.2)	59 (1.7)
J01DB First-generation cephalosporins (cephalexin, cefadroxil, cephradine, and cefazolin)	2 (0.1)	7 (0.7)	39 (4.9)	2 (0.3)	11 (3.4)	385 (11.2)
J01CF Beta-lactamase-resistant penicillins (oxacillin)	5 (0.3)	0 (0)	38 (4.7)	9 (1.4)	0 (0)	22 (0.6)
J01EE Combinations of sulfonamides and trimethoprim, including derivatives (trimethoprim-sulfamethoxazole)	32 (2.2)	3 (0.3)	5 (0.6)	8 (1.2)	1 (0.3)	134 (3.9)
J01XB Polymyxins (colistin)	29 (2)	4 (0.4)	2 (0.2)	6 (0.9)	3 (0.9)	18 (0.5)
J01DC Second-generation cephalosporins (cefuroxime)	1 (0.1)	2 (0.2)	2 (0.2)	0 (0)	8 (2.4)	29 (0.8)
J01AA Tetracyclines (doxycycline)	7 (0.5)	0 (0)	1 (0.1)	0 (0)	0 (0)	16 (0.5)
J01XE Nitrofuran derivatives (nitrofurantoin)	0 (0)	0 (0)	0 (0)	0 (0)	0 (0)	10 (0.3)
J01CE Beta-lactamase-sensitive penicillins (penamecillin)	0 (0)	0 (0)	0 (0)	0 (0)	0 (0)	0 (0)
Other antibiotics	50 (3.4)	36 (3.5)	49 (6.1)	19 (2.9)	11 (3.4)	228 (6.7)
**Total**	1477	1043	803	657	328	3427

^a^: Number of antibiotic prescriptions for the 5310 patients who were included in the PPS.

**Table 5:** Compliance with guidelines for treatment and prophylaxis prescriptions.

	Total		Chile		Colombia		Mexico		Peru		Panama	
	AB Prescribed *n*	Compliance *n* (%)	AB Prescribed *n*	Compliance *n* (%)	AB Prescribed *n*	Compliance *n* (%)	AB Prescribed *n*	Compliance *n* (%)	AB Prescribed *n*	Compliance *n* (%)	AB Prescribed *n*	Compliance *n* (%)
**Guidelines compliance**	7735	4784 (61.8)	1927	1417 (73.5)	645	456 (70.7)	1790	1013 (56.6)	3113	1741 (55.9)	260	157 (60.4)
**Adult medical wards**	2028	1271 (62.7)	600	440 (73.3)	123	97 (78.9)	466	249 (53.4)	839	485 (57.8)	0	0 (0.0)
**Adult surgical wards**	1487	729 (49.0)	458	307 (67.0)	47	34 (72.3)	518	213 (41.1)	464	175 (37.7)	0	0 (0.0)
**Intensive care units**	1345	952 (70.8)	427	325 (76.1)	132	105 (79.5)	248	185 (74.6)	447	290 (64.9)	91	47 (51.6)
**Pediatric wards**	1575	1103 (70.0)	220	173 (78.6)	18	16 (88.9)	454	338 (74.4)	714	466 (65.3)	169	110 (65.1)
**Obstetrics and gynecology**	393	181 (46.1)	94	73 (77.7)	32	13 (40.6)	75	14 (18.7)	192	81 (42.2)	0	0 (0.0)
**Adult high-risk units**	158	125 (79.1)	57	47 (82.5)	22	21 (95.5)	4	2 (50.0)	75	55 (73.3)	0	0 (0.0)
**Mixed wards**	749	423 (56.5)	71	52 (73.2)	271	170 (62.7)	25	12 (48.0)	382	189 (49.5)	0	0 (0.0)

**Table 6:** Factors associated with the prevalence of antibiotic use in selected hospitals (2022–2023, *n*: 11,094).

		Univariate Analysis		Multivariate Analysis	
	*n*	OR IC 95%	*p* value	OR IC 95%	*p* value
Intercept	-			0.19 (0.12–0.29)	<0.001
**Age**					
Less than 1 year	1487	1		1	
1–4 years	679	1.67 (1.39–2.01)	<0.001	1.69 (1.36–2.1)	<0.001
5–17 years	1099	1.61 (1.37–1.89)	<0.001	1.60 (1.32–1.92)	<0.001
18–65 years	5149	1.75 (1.54–1.98)	<0.001	1.99 (1.6–2.48)	<0.001
More than 65 years	2677	1.83 (1.60–2.10)	<0.001	1.84 (1.46–2.31)	<0.001
**Gender**					
Female	5624	1		1	
Male	5453	1.17 (1.09–1.26)	<0.001	1.05 (0.96–1.15)	0.296
Transgender	11	0.75 (0.22–2.53)	0.642	0.65 (0.14–3.01)	0.582
**Ward**					
Adult high-risk units	247	1		1	
Adult medical	3303	0.83 (0.64–1.08)	0.162	0.89 (0.67–1.18)	0.400
Adult surgical	1792	1.30 (0.99–1.70)	0.056	1.51 (1.12–2.02)	0.007
Intensive care units	1493	1.40 (1.07–1.84)	0.015	1.45 (1.05–1.99)	0.022
Mixed	1006	1.29 (0.97–1.71)	0.078	1.49 (1.1–2.02)	0.01
Obstetrics and gynecology	893	0.52 (0.39–0.69)	<0.001	0.69 (0.5–0.95)	0.023
Pediatric	2360	0.70 (0.54–0.92)	0.010	1.08 (0.76–1.52)	0.675
**Previous history of hospitalization (within 90 days)**					
No	7464	1		1	
Yes	1006	1.54 (1.35–1.76)	<0.001	1.47 (1.27–1.69)	<0.001
**Patients with a catheter**					
No	1951	1		1	
Yes	9127	3.17 (2.83–3.54)	<0.001	2.71 (2.37–3.09)	<0.001
**Patients intubated**					
No	8899	1		1	
Yes	2153	1.92 (1.74–2.12)	<0.001	1.53 (1.35–1.74)	<0.001

## Data Availability

The original contributions presented in this study are included in the article/Supplementary Material. Further inquiries can be directed to the corresponding author.

## Supplementary Materials

The following supporting information can be downloaded at: https://www.mdpi.com/article/10.3390/antibiotics14111078/s1, File S1: PPS data collection form.
