# Supplementary material for: Point Prevalence Survey of Antibiotic Use in Latin American Hospitals: 2022–2023
Source: Antibiotics (Basel). 2025 Oct 27;14(11):1078. doi: 10.3390/antibiotics14111078 (PMC13420492; doi:10.3390/antibiotics14111078)
Supplement: Supplementary file 1 [file antibiotics-14-01078-s001.zip › antibiotics-3912915-supplementary.pdf]

# PPS data collection form

This is the PAHO/WHO Point Prevalence survey for antibiotic use in hospitals.

| | |
|---|---|
| Hospital Name | ◯ Victoria Hospital ◯ St. Jude Hospital |
| Data collector (team) No. | ◯ 1 ◯ 2 ◯ 3 ◯ 4<br>◯ 5 ◯ 6 |

## Patient data

| | |
|---|---|
| Date of survey | ______________________ |
| Type of Ward | ◯ Paediatric medical ward<br>◯ Paediatric surgical ward<br>◯ High risk paediatric ward<br>◯ Paediatric intensive care unit<br>◯ Neonatal medical ward<br>◯ Neonatal intensive care unit<br>◯ Adult medical ward<br>◯ Adult surgical ward<br>◯ High risk adult ward<br>◯ Adult intensive care unit<br>◯ Mixed ward<br>◯ Obstetrics and gynecology<br>(High risk units are defined as units or wards that by the type of care they provide are high consumers of antibiotics. The usual therapy for patients in these high risk units often require antibiotics as part of their care. High risk units consist of wards with the following specialties: Haematology, Oncology, Burns, Transplantation and Infectious Diseases ) |

| | |
|---|---|
| Ward specialty (For GPHC only) | ◯ Male Medical 1<br>◯ Male Surgical 1<br>◯ Male Medical 2<br>◯ Male Surgical 2<br>◯ Ophthalmology<br>◯ Male HDU Medical<br>◯ Male HDU Surgical<br>◯ Female Medical 1<br>◯ Female Surgical 1<br>◯ Female Medical 2<br>◯ Female Surgical 2<br>◯ Female HDU Medical<br>◯ Female HDU Surgical<br>◯ Infectious Disease Ward (IDW)<br>◯ Psychiatric<br>◯ Maternity<br>◯ Gynecology<br>◯ NICU<br>◯ Step down NICU<br>◯ Post natal<br>◯ BCU<br>◯ Pediatric Medical<br>◯ Pediatric Surgical<br>◯ Pediatric DHU<br>◯ ICU<br>◯ CICU |
| Ward specialty (For Victoria Hospital only) | ◯ Accident and Emergency<br>◯ Chest Wing<br>◯ Acute Medical Unit<br>◯ Obstetrics<br>◯ Pediatrics<br>◯ Neonatal Unit<br>◯ Female Surgical Unit<br>◯ Gynaecology<br>◯ Male Surgical Unit<br>◯ Operating Theatre<br>◯ Intensive Care Unit<br>◯ Day Surgery Unit<br>◯ Out-Patients Clinic<br>◯ Dialysis Unit |
| Ward specialty (For St. Jude Hospital only) | ◯ Surgical ward<br>◯ Medical Ward<br>◯ Maternity (Newborn rooms with the mother)<br>◯ Pediatrics<br>◯ ICU<br>◯ Nursery<br>◯ ER Holding Area |
| Patient's Registration Number | ______________________________<br>(If the infant registration number is the same as the mother, please use the mother's registration number and add "IO" at the end) |
| Gender | ◯ Male<br>◯ Female<br>◯ Transgender<br>◯ Unknown |

| | |
|---|---|
| Date of birth available? | ◯ Yes<br>◯ No |
| Date of Birth (Y-M-D) | __________________ |
| Age (years) | __________________ |
| Age of the patient | __________________ |
| Preterm birth | ◯ Yes ◯ No ◯ Unknown |
| Preterm birth | ◯ Yes ◯ No ◯ Unknown |
| Children's weight (kg) | __________________ |
| Children's weight (kg) | __________________ |
| Date of admission | __________________ |
| Any catheterization? | ◯ Yes ◯ No ◯ Unknown |
| Type of catheterization | ☐ Urinary<br>☐ Peripheral<br>☐ Central<br>☐ Peritoneal<br>☐ Haemodialysis<br>☐ Other<br>☐ Unknown |
| Any intubation? | ◯ Yes ◯ No ◯ Unknown |
| The patient has malaria? | ◯ Yes ◯ No ◯ Unknown |
| The patient has tuberculosis? | ◯ Yes ◯ No ◯ Unknown |
| The patient has HIV? | ◯ Yes ◯ No ◯ Unknown |
| CD4 count in the past 6 months. | __________________<br>(Specify in cells/mm3. If unknown, state "Unknown") |
| The patient is malnourished? | ◯ Yes ◯ No ◯ Unknown |
| Is the patient transferred in from another health facility? | ◯ Yes ◯ No ◯ Unknown |
| Is the patient transferred in from another patient unit at the hospital? | ◯ Yes ◯ No ◯ Unknown |

| | |
|---|---|
| Name of the unit | ______________________<br>(State "unknown" if unknown) |
| Has the patient been hospitalized at the hospital surveyed within 90 days before current admission? | ◯ Yes ◯ No ◯ Unknown |
| Was hospitalization as a result of birth? | ◯ Yes ◯ No ◯ Unknown<br>(e.g. patients was hospitalized because they were born in this hospital ) |
| Is the patient currently on antibiotic treatment other than TB drug? | ◯ Yes ◯ No |

## Indication No.1

| | |
|---|---|
| Patient acquired infection from... | ◯ Hospital Associated Infection (HAI)<br>◯ Community Acquired Infection (CAI)<br>◯ Home Based Care or long-term community care facilities<br>◯ Unknown<br>◯ Other |

| | |
|---|---|
| Diagnosis of indication No.1 | ◯ CNS (Infections of the central nervous system)<br>◯ EYE (Endophthalmitis)<br>◯ ENT (Infections of ear, nose, throat, larynx and mouth)<br>◯ BRON (Acute bronchitis or exacerbations of chronic bronchitis)<br>◯ PNEU (Pneumonia)<br>◯ CF (Cystic Fibrosis)<br>◯ CVS (Cardiovascular infections: endocarditis, vascular graft)<br>◯ GI (Gastrointestinal infections (e.g. salmonellosis, antibiotic-associated diarrhoea))<br>◯ IA (Intra-abdominal sepsis, including hepatobiliary)<br>◯ SST-SSI (Surgical site infection involving skin or soft tissue but not bone)<br>◯ SST-O (Cellulitis, wound, deep soft tissue not involving bone, not related to surgery)<br>◯ BJ-SSI (Septic arthritis, osteomyelitis of surgical site)<br>◯ BJ-O (Septic arthritis, osteomyelitis, not related to surgery)<br>◯ CYS (Symptomatic lower urinary tract infection (e.g. cystitis))<br>◯ PYE (Symptomatic upper urinary tract infection (e.g. pyelonephritis))<br>◯ ASB (Asymptomatic bacteriuria)<br>◯ OBGY (Obstetric or gynaecological infections, STD in women)<br>◯ GUM (Prostatitis, epididymo-orchitis, STD in men)<br>◯ BAC (Laboratory-confirmed bacteraemia)<br>◯ CSEP (Clinical sepsis (suspected bloodstream infection without lab confirmation/results are not available, no blood cultures collected or negative blood culture), excluding febrile neutropenia)<br>◯ FN (Febrile neutropenia or other form of manifestation of infection in immunocompromised host (e.g. HIV, chemotherapy, etc.) with no clear anatomical site)<br>◯ SIRS (Systemic inflammatory response with no clear anatomical site)<br>◯ UND (Completely undefined; site with no systemic inflammation)<br>◯ NA (Not applicable; for antibiotic use other than treatment)<br>◯ Unknown<br>◯ Other |
| If diagnosis is not available from the list above. Please state here. | __________________________________ |
| Is it a prophylaxis or treatment | ◯ Prophylaxis<br>◯ Treatment<br>◯ Both<br>◯ Unknown |
| Is it a medical or surgical prophylaxis | ◯ Medical prophylaxis<br>◯ Surgical prophylaxis<br>◯ Both<br>◯ Unknown |

| | |
|---|---|
| Duration of surgical prophylaxis | ◯ One dose<br>◯ Multiple doses on one day<br>◯ Multiple doses on more than one day<br>◯ Unknown |
| Is it an empiric or targeted therapy? | ◯ Empiric therapy<br>◯ Targeted therapy<br>◯ Unknown<br>(Empiric means blind therapy; Targeted therapy means antibiotic was chosen or consolidated after Culture & Sensitivity results) |
| Start date of the first antibiotics was administered for this indication (Y-M-D) | ______________________________<br>(The day on which the first dose of the first antibiotics was administered for this indication) |
| Has a sample been taken for microbiology diagnostic? | ◯ Yes ◯ No ◯ Unknown |
| Culture and Sensitivity Test results were available in documentation? | ◯ Yes ◯ No ◯ Unknown |
| Name of the bacteria isolated in laboratory | ______________________________<br>(If unknown, state as "unknown") |
| Type of antimicrobial resistance | ☐ carbapenem resistance<br>☐ Extended-spectrum beta-lactamases (ESBL)<br>☐ methicillin resistance<br>☐ colistin resistance<br>☐ vancomycin resistance<br>☐ Unknown<br>☐ Other resistance<br>☐ No resistance |

## Indication No.2

| | |
|---|---|
| Is there a second indication? | ◯ Yes ◯ No |
| Patient acquired infection from... | ◯ Hospital Associated Infection (HAI)<br>◯ Community Acquired Infection (CAI)<br>◯ Home Based Care or long-term community care facilities<br>◯ Unknown<br>◯ Other |

| | |
|---|---|
| Diagnosis of indication No.2 | ◯ CNS (Infections of the central nervous system)<br>◯ EYE (Endophthalmitis)<br>◯ ENT (Infections of ear, nose, throat, larynx and mouth)<br>◯ BRON (Acute bronchitis or exacerbations of chronic bronchitis)<br>◯ PNEU (Pneumonia)<br>◯ CF (Cystic Fibrosis)<br>◯ CVS (Cardiovascular infections: endocarditis, vascular graft)<br>◯ GI (Gastrointestinal infections (e.g. salmonellosis, antibiotic-associated diarrhoea))<br>◯ IA (Intra-abdominal sepsis, including hepatobiliary)<br>◯ SST-SSI (Surgical site infection involving skin or soft tissue but not bone)<br>◯ SST-O (Cellulitis, wound, deep soft tissue not involving bone, not related to surgery)<br>◯ BJ-SSI (Septic arthritis, osteomyelitis of surgical site)<br>◯ BJ-O (Septic arthritis, osteomyelitis, not related to surgery)<br>◯ CYS (Symptomatic lower urinary tract infection (e.g. cystitis))<br>◯ PYE (Symptomatic upper urinary tract infection (e.g. pyelonephritis))<br>◯ ASB (Asymptomatic bacteriuria)<br>◯ OBGY (Obstetric or gynaecological infections, STD in women)<br>◯ GUM (Prostatitis, epididymo-orchitis, STD in men)<br>◯ BAC (Laboratory-confirmed bacteraemia)<br>◯ CSEP (Clinical sepsis (suspected bloodstream infection without lab confirmation/results are not available, no blood cultures collected or negative blood culture), excluding febrile neutropenia)<br>◯ FN (Febrile neutropenia or other form of manifestation of infection in immunocompromised host (e.g. HIV, chemotherapy, etc.) with no clear anatomical site)<br>◯ SIRS (Systemic inflammatory response with no clear anatomical site)<br>◯ UND (Completely undefined; site with no systemic inflammation)<br>◯ NA (Not applicable; for antibiotic use other than treatment)<br>◯ Unknown<br>◯ Other |
| If diagnosis is not available from the list above. Please state here. | __________________________________ |
| Is it a prophylaxis or treatment | ◯ Prophylaxis<br>◯ Treatment<br>◯ Both<br>◯ Unknown |
| Is it a medical or surgical prophylaxis | ◯ Medical prophylaxis<br>◯ Surgical prophylaxis<br>◯ Both<br>◯ Unknown |

| | |
|---|---|
| Duration of surgical prophylaxis | ◯ One dose<br>◯ Multiple doses on one day<br>◯ Multiple doses on more than one day<br>◯ Unknown |
| Is it an empiric or targeted therapy? | ◯ Empiric therapy<br>◯ Targeted therapy<br>◯ Unknown<br>(Empiric means blind therapy; Targeted therapy means antibiotic was chosen or consolidated after Culture & Sensitivity results) |
| Start date of the first antibiotics was administered for this indication (Y-M-D) | __________________________________<br>(The day on which the first dose of the first antibiotics was administered for this indication) |
| Has a sample been taken for microbiology diagnostic? | ◯ Yes ◯ No ◯ Unknown |
| Culture and Sensitivity Test results were available in documentation? | ◯ Yes ◯ No ◯ Unknown |
| Name of the bacteria isolated in laboratory | __________________________________<br>(If unknown, state as "unknown") |
| Type of antimicrobial resistance | ☐ carbapenem resistance<br>☐ Extended-spectrum beta-lactamases (ESBL)<br>☐ methicillin resistance<br>☐ colistin resistance<br>☐ vancomycin resistance<br>☐ Unknown<br>☐ Other resistance<br>☐ No resistance |

## Indication No.3

| | |
|---|---|
| Is there a third indication? | ◯ Yes ◯ No |
| Patient acquired infection from.. | ◯ Hospital Associated Infection (HAI)<br>◯ Community Acquired Infection (CAI)<br>◯ Home Based Care or long-term community care facilities<br>◯ Unknown<br>◯ Other |

| | |
|---|---|
| Diagnosis of indication No.3 | ◯ CNS (Infections of the central nervous system)<br>◯ EYE (Endophthalmitis)<br>◯ ENT (Infections of ear, nose, throat, larynx and mouth)<br>◯ BRON (Acute bronchitis or exacerbations of chronic bronchitis)<br>◯ PNEU (Pneumonia)<br>◯ CF (Cystic Fibrosis)<br>◯ CVS (Cardiovascular infections: endocarditis, vascular graft)<br>◯ GI (Gastrointestinal infections (e.g. salmonellosis, antibiotic-associated diarrhoea))<br>◯ IA (Intra-abdominal sepsis, including hepatobiliary)<br>◯ SST-SSI (Surgical site infection involving skin or soft tissue but not bone)<br>◯ SST-O (Cellulitis, wound, deep soft tissue not involving bone, not related to surgery)<br>◯ BJ-SSI (Septic arthritis, osteomyelitis of surgical site)<br>◯ BJ-O (Septic arthritis, osteomyelitis, not related to surgery)<br>◯ CYS (Symptomatic lower urinary tract infection (e.g. cystitis))<br>◯ PYE (Symptomatic upper urinary tract infection (e.g. pyelonephritis))<br>◯ ASB (Asymptomatic bacteriuria)<br>◯ OBGY (Obstetric or gynaecological infections, STD in women)<br>◯ GUM (Prostatitis, epididymo-orchitis, STD in men)<br>◯ BAC (Laboratory-confirmed bacteraemia)<br>◯ CSEP (Clinical sepsis (suspected bloodstream infection without lab confirmation/results are not available, no blood cultures collected or negative blood culture), excluding febrile neutropenia)<br>◯ FN (Febrile neutropenia or other form of manifestation of infection in immunocompromised host (e.g. HIV, chemotherapy, etc.) with no clear anatomical site)<br>◯ SIRS (Systemic inflammatory response with no clear anatomical site)<br>◯ UND (Completely undefined; site with no systemic inflammation)<br>◯ NA (Not applicable; for antibiotic use other than treatment)<br>◯ Unknown<br>◯ Other |
| If diagnosis is not available from the list above. Please state here. | __________________________________ |
| Is it a prophylaxis or treatment | ◯ Prophylaxis<br>◯ Treatment<br>◯ Both<br>◯ Unknown |
| Is it a medical or surgical prophylaxis | ◯ Medical prophylaxis<br>◯ Surgical prophylaxis<br>◯ Both<br>◯ Unknown |

| | |
|---|---|
| Duration of surgical prophylaxis | ◯ One dose<br>◯ Multiple doses on one day<br>◯ Multiple doses on more than one day<br>◯ Unknown |
| Is it an empiric or targeted therapy? | ◯ Empiric therapy<br>◯ Targeted therapy<br>◯ Unknown<br>(Empiric means blind therapy; Targeted therapy means antibiotic was chosen or consolidated after Culture & Sensitivity results) |
| Start date of the first antibiotics was administered for this indication (Y-M-D) | __________________________________<br>(The day on which the first dose of the first antibiotics was administered for this indication) |
| Has a sample been taken for microbiology diagnostic? | ◯ Yes ◯ No ◯ Unknown |
| Culture and Sensitivity Test results were available in documentation? | ◯ Yes ◯ No ◯ Unknown |
| Name of the bacteria isolated in laboratory | __________________________________<br>(If unknown, state as "unknown") |
| Type of antimicrobial resistance | ☐ carbapenem resistance<br>☐ Extended-spectrum beta-lactamases (ESBL)<br>☐ methicillin resistance<br>☐ colistin resistance<br>☐ vancomycin resistance<br>☐ Unknown<br>☐ Other resistance<br>☐ No resistance |

## Antibiotic No.1

| | |
|---|---|
| Related indication number | __________________________________<br>(Please enter the No. value of the related indication entered in the earlier section. If the antibiotic cannot be linked to one indication, please enter "No". If more than one indication are linked to the antibiotic, specify the counter values of the linked indications separated (e.g. 1,2)) |
| Start date of antibiotic (Y-M-D) | __________________________________ |
| Is it a combination product? | ◯ Yes ◯ No<br>(Combination products are products containing two antibiotic substances or an antibiotic substance and an enzyme inhibitor) |

| | |
|---|---|
| Name of each antibiotic substance in the combination product | ______________________________<br>(For combination product, entered each antibiotic substance name seperated with comma (e.g. trimethoprim, sulfamethoxazole). Enter "No" if it's not possible to get the information.) |
| Doses of each of active substances in the combination product | ______________________________<br>(The dose of each substance including the enzyme inhibitors should be entered in the same order as in the antibiotic name in the last question (e.g. 160mg, 800mg). Enter "No" if it's not possible to get the information.) |
| Name of the antibiotic in patient's note | ______________________________ |
| Unit dose administered to the patient | ______________________________ |
| Measurement unit of the unit dose | ◯ g (gram)<br>◯ mg (milligram)<br>◯ IU (international unit)<br>◯ MU (millions of international units) |
| Daily frequency of the administration of the unit dose | ______________________________<br>(e.g. amoxicillin treatment given every 8 hours. Daily frequency = 24 hours /8 hours=3) |
| Please leave comment here if you are not sure about the calculation. | ______________________________ |
| Route of administration of the antibiotic | ◯ Oral<br>◯ Parenteral<br>◯ Inhalation<br>◯ Rectal |
| Type of IV | ◯ IV-B: intermittent<br>◯ IV-C: continuous<br>◯ IV-E: extended<br>◯ Other |
| Is the oral antibiotic is the result from a switch from IV to oral ? | ◯ Yes ◯ No ◯ Unknown |
| Type of prescriber | ◯ Specialist<br>◯ Medical Officer<br>◯ Nurse<br>◯ Resident |
| Number of missed doses | ______________________________<br>(The count from the start date of the current antibiotic till the current date of how many doses were missed and state it a simple count. If unknown, specify Unknown) |

Reason for missing dose (if applicable)

______________________________
(State "unknown" if unknown)

## Antibiotic No.2

Is there a second antibiotic?

◯ Yes ◯ No

Related indication number

______________________________
(Please enter the No. value of the related indication entered in the earlier section. If the antibiotic cannot be linked to one indication, please enter "No". If more than one indication are linked to the antibiotic, specify the counter values of the linked indications separated (e.g. 1,2))

Start date of antibiotic (Y-M-D)

______________________________

Is it a combination product?

◯ Yes ◯ No
(Combination products are products containing two antibiotic substances or an antibiotic substance and an enzyme inhibitor)

Name of each antibiotic substance in the combination product

______________________________
(For combination product, entered each antibiotic substance name seperated with comma (e.g. trimethoprim, sulfamethoxazole). Enter "No" if it's not possible to get the information.)

Doses of each of active substances in the combination product

______________________________
(The dose of each substance including the enzyme inhibitors should be entered in the same order as in the antibiotic name in the last question (e.g. 160mg, 800mg). Enter "No" if it's not possible to get the information.)

Name of the antibiotic in patient's note

______________________________

Unit dose administered to the patient

______________________________

Measurement unit of the unit dose

◯ g (gram)
◯ mg (milligram)
◯ IU (international unit)
◯ MU (millions of international units)

Daily frequency of the administration of the unit dose

______________________________
(e.g. amoxicillin treatment given every 8 hours. Daily frequency = 24 hours /8 hours=3 )

| | |
|---|---|
| Please leave comment here if you are not sure about the calculation. | ________________________ |
| Route of administration of the antibiotic | ◯ Oral<br>◯ Parenteral<br>◯ Inhalation<br>◯ Rectal |
| Type of IV | ◯ IV-B: intermittent<br>◯ IV-C: continuous<br>◯ IV-E: extended<br>◯ Other |
| Is the oral antibiotic is the result from a switch from IV to oral ? | ◯ Yes ◯ No ◯ Unknown |
| Type of prescriber | ◯ Specialist<br>◯ Medical Officer<br>◯ Nurse<br>◯ Resident |
| Number of missed doses | ________________________<br>(The count from the start date of the current antibiotic till the current date of how many doses were missed and state it a simple count. If unknown, specify Unknown) |
| Reason for missing dose (if applicable) | ________________________<br>(State "unknown" if unknown) |

## Antibiotic No.3

| | |
|---|---|
| Is there a third antibiotic? | ◯ Yes ◯ No |
| Related indication number | ________________________<br>(Please enter the No. value of the related indication entered in the earlier section. If the antibiotic cannot be linked to one indication, please enter "No". If more than one indication are linked to the antibiotic, specify the counter values of the linked indications separated (e.g. 1,2)) |
| Start date of antibiotic (Y-M-D) | ________________________ |
| Is it a combination product? | ◯ Yes ◯ No<br>(Combination products are products containing two antibiotic substances or an antibiotic substance and an enzyme inhibitor) |

| | |
|---|---|
| Name of each antibiotic substance in the combination product | __________________________________ (For combination product, entered each antibiotic substance name seperated with comma (e.g. trimethoprim, sulfamethoxazole). Enter "No" if it's not possible to get the information.) |
| Doses of each of active substances in the combination product | __________________________________ (The dose of each substance including the enzyme inhibitors should be entered in the same order as in the antibiotic name in the last question (e.g. 160mg, 800mg). Enter "No" if it's not possible to get the information.) |
| Name of the antibiotic in patient's note | __________________________________ |
| Unit dose administered to the patient | __________________________________ |
| Measurement unit of the unit dose | ◯ g (gram)<br>◯ mg (milligram)<br>◯ IU (international unit)<br>◯ MU (millions of international units) |
| Daily frequency of the administration of the unit dose | __________________________________ (e.g. amoxicillin treatment given every 8 hours. Daily frequency = 24 hours /8 hours=3) |
| Please leave comment here if you are not sure about the calculation. | __________________________________ |
| Route of administration of the antibiotic | ◯ Oral<br>◯ Parenteral<br>◯ Inhalation<br>◯ Rectal |
| Type of IV | ◯ IV-B: intermittent<br>◯ IV-C: continuous<br>◯ IV-E: extended<br>◯ Other |
| Is the oral antibiotic is the result from a switch from IV to oral ? | ◯ Yes ◯ No ◯ Unknown |
| Type of prescriber | ◯ Specialist<br>◯ Medical Officer<br>◯ Nurse<br>◯ Resident |
| Number of missed doses | __________________________________ (The count from the start date of the current antibiotic till the current date of how many doses were missed and state it a simple count. If unknown, specify Unknown) |

Reason for missing dose (if applicable)

__________________________________
(State "unknown" if unknown)

## Antibiotic No.4

Is there a fourth antibiotic? ◯ Yes ◯ No

Related indication number

__________________________________
(Please enter the counter value of the related indication entered earlier. If the antibiotic cannot be linked to one indication, please enter "No". If more than one indication are linked to the antibiotic, specify the counter values of the linked indications separated (e.g. 1,2))

Start date of antibiotic (Y-M-D)

__________________________________

Is it a combination product? ◯ Yes ◯ No
(Combination products are products containing two antibiotic substances or an antibiotic substance and an enzyme inhibitor)

Name of each antibiotic substance in the combination product

__________________________________
(For combination product, entered each antibiotic substance name seperated with comma (e.g. trimethoprim, sulfamethoxazole). Enter "No" if it's not possible to get the information.)

Doses of each of active substances in the combination product

__________________________________
(The dose of each substance including the enzyme inhibitors should be entered in the same order as in the antibiotic name in the last question (e.g. 160mg, 800mg). Enter "No" if it's not possible to get the information.)

Name of the antibiotic in patient's note

__________________________________

Unit dose administered to the patient

__________________________________

Measurement unit of the unit dose

◯ g (gram)
◯ mg (milligram)
◯ IU (international unit)
◯ MU (millions of international units)

Daily frequency of the administration of the unit dose

__________________________________
(e.g. amoxicillin treatment given every 8 hours. Daily frequency = 24/8=3)

Please leave comment here if you are not sure about the calculation.

__________________________________

| | |
|---|---|
| Route of administration of the antibiotic | ◯ Oral<br>◯ Parenteral<br>◯ Inhalation<br>◯ Rectal |
| Type of IV | ◯ IV-B: intermittent<br>◯ IV-C: continuous<br>◯ IV-E: extended<br>◯ Other |
| Is the oral antibiotic is the result from a switch from IV to oral ? | ◯ Yes ◯ No ◯ Unknown |
| Type of prescriber | ◯ Specialist<br>◯ Medical Officer<br>◯ Nurse<br>◯ Resident |
| Number of missed doses | __________________________________<br>(The count from the start date of the current antibiotic till the current date of how many doses were missed and state it a simple count. If unknown, specify Unknown) |
| Reason for missing dose (if applicable) | __________________________________<br>(State "unknown" if unknown) |

## Antibiotic No.5

| | |
|---|---|
| Is there a fifth antibiotic? | ◯ Yes ◯ No |
| Related indication number | __________________________________<br>(Please enter the No. value of the related indication entered in the earlier section. If the antibiotic cannot be linked to one indication, please enter "No". If more than one indication are linked to the antibiotic, specify the counter values of the linked indications separated (e.g. 1,2)) |
| Start date of antibiotic (Y-M-D) | __________________________________ |
| Is it a combination product? | ◯ Yes ◯ No<br>(Combination products are products containing two antibiotic substances or an antibiotic substance and an enzyme inhibitor) |
| Name of each antibiotic substance in the combination product | __________________________________<br>(For combination product, entered each antibiotic substance name seperated with comma (e.g. trimethoprim, sulfamethoxazole). Enter "No" if it's not possible to get the information.) |

| | |
|---|---|
| Doses of each of active substances in the combination product | ____________________<br>(The dose of each substance including the enzyme inhibitors should be entered in the same order as in the antibiotic name in the last question (e.g. 160mg, 800mg). Enter "No" if it's not possible to get the information.) |
| Name of the antibiotic in patient's note | ____________________ |
| Unit dose administered to the patient | ____________________ |
| Measurement unit of the unit dose | ◯ g (gram)<br>◯ mg (milligram)<br>◯ IU (international unit)<br>◯ MU (millions of international units) |
| Daily frequency of the administration of the unit dose | ____________________<br>(e.g. amoxicillin treatment given every 8 hours. Daily frequency = 24/8=3) |
| Please leave comment here if you are not sure about the calculation. | ____________________ |
| Route of administration of the antibiotic | ◯ Oral<br>◯ Parenteral<br>◯ Inhalation<br>◯ Rectal |
| Type of IV | ◯ IV-B: intermittent<br>◯ IV-C: continuous<br>◯ IV-E: extended<br>◯ Other |
| Is the oral antibiotic is the result from a switch from IV to oral ? | ◯ Yes ◯ No ◯ Unknown |
| Type of prescriber | ◯ Specialist<br>◯ Medical Officer<br>◯ Nurse<br>◯ Resident |
| Number of missed doses | ____________________<br>(The count from the start date of the current antibiotic till the current date of how many doses were missed and state it a simple count. If unknown, specify Unknown) |
| Reason for missing dose (if applicable) | ____________________<br>(State "unknown" if unknown) |

## Notes

Other comments
